# Supplementary material for: Genomic insights into the evolutionary relationships and demographic history of kiwi
Source: PLoS One. 2022 Oct 10;17(10):e0266430. doi: 10.1371/journal.pone.0266430 (PMC9550048; doi:10.1371/journal.pone.0266430)
Supplement: S1 File — (DOCX) [file pone.0266430.s001.docx]

**Supporting information**

**Supplementary table S1: Estimated population sizes and conservation statuses of the five extant kiwi species.** DOC - Department of conservation of New Zealand. IUCN - International Union of Conservation of Nature. For southern brown kiwi, DOC conservation statuses vary for different populations, and include nationally critical, nationally vulnerable, and nationally endangered.

| **Species** | **DOC status** | **IUCN status** |
| --- | --- | --- |
| Great spotted kiwi | Nationally vulnerable | Vulnerable |
| Little spotted kiwi | At risk - recovering | Near threatened |
| Okarito brown kiwi | Nationally vulnerable | Vulnerable |
| Southern brown kiwi | Population dependent | Vulnerable |
| North Island brown kiwi | At risk -declining | Vulnerable |

**Supplementary table S2:** Accession codes for the raw read and genome assemblies of all species included in this study.

| Common name | Species | Read accession | Assembly | Reference |
| --- | --- | --- | --- | --- |
| Southern Cassowary | *Casuarius casuarius* | SRR6918130, SRR6918128 | GCA_003342895.1 | [14] |
| Emu | *Dromaius novaehollandiae* | SRR6918125, SRR6918126 | GCF_003342905.1 | [14] |
| Ostrich | *Struthio camelus* | SRR950909, SRR950910, SRR950911 | GCF_000698965.1 | [16] |
| Greater Rhea | *Rhea americana* | SRR6918111, SRR6918146 | GCA_ 003343005.1 | [14] |
| Great spotted kiwi | *Apteryx maxima* | SRR6918112 | GCA_003342985.1 | [14] |
| North Island brown kiwi | *Apteryx mantelli* | ERR519283, ERR519284 | GCF_001039765.1 | [[2]](https://paperpile.com/c/pUvd6E/erlQ+5Vnx) |
| Southern brown kiwi | *Apteryx australis* | SRR15100622 | NA | [[15]](https://paperpile.com/c/pUvd6E/cl6z) |
| Little spotted kiwi | *Apteryx owenii* | SRR6918114, SRR6918115 | GCA_003342965.1 | [14] |
| Okarito brown kiwi | *Apteryx rowi* | SRR6918118 | GCF_003343035.1 | [14] |

**Supplementary table S3:** Assembly statistics for all assemblies used in this study. GSK - Great spotted kiwi, NIBK - North Island brown kiwi, LSK - Little spotted kiwi, OBK - Okarito brown kiwi, SBK - Southern brown kiwi. N50 is the length of the shortest scaffold when summing the length of the fewest scaffolds possible to make up 50% of the total assembly length. L75 is the smallest number of scaffolds whose length sum to 75% of the genome’s size.

| **Assembly** | **Length (bp)** | **N50** | **Largest contig (bp)** | **L75** |
| --- | --- | --- | --- | --- |
| Emu | 1,192,254,075 | 3,305,683 | 11,235,192 | 238 |
| GSK | 1,221,441,152 | 1,371,329 | 7,925,965 | 553 |
| NIBK | 1,523,972,539 | 5,679,020 | 63,182,071 | 277 |
| OBK | 1,228,902,913 | 1,672,276 | 12,705,442 | 456 |
| LSK | 1,231,276,503 | 1,622,467 | 17,302,385 | 485 |
| Cassowary | 1,209,001,349 | 3,704,890 | 15,094,948 | 210 |
| Ostrich | 1,225,041,896 | 3,641,246 | 19,375,980 | 221 |
| Rhea | 1,160,836,514 | 3,949,999 | 21,551,348 | 181 |

**Supplementary table S4:** Results of all mappings performed in this study. GSK - Great spotted kiwi, NIBK - North Island brown kiwi, LSK - Little spotted kiwi, OBK - Okarito brown kiwi, SBK - Southern brown kiwi.

| **Species** | **Reference** | **Conspecific reference?** | **# of reads mapping** | **Average coverage** |
| --- | --- | --- | --- | --- |
| Cassowary | Cassowary | Yes | 452,608,345 | 42.69 |
| Emu | Emu | Yes | 341,640,306 | 38.89 |
| GSK | GSK | Yes | 241,363,500 | 24.52 |
| GSK | Emu | No | 215,253,668 | 19.25 |
| NIBK | Emu | No | 213,749,849 | 17.73 |
| NIBK | GSK | No | 273,984,072 | 22.19 |
| NIBK | NIBK | Yes | 253,716,571 | 20.86 |
| Ostrich | Ostrich | Yes | 461,823,968 | 38.71 |
| LSK | Emu | No | 242,035,001 | 25.86 |
| LSK | GSK | No | 303,699,167 | 31.50 |
| LSK | NIBK | No | 277,648,053 | 29.70 |
| LSK | LSK | Yes | 308,322,696 | 31.67 |
| Rhea | Rhea | Yes | 479,748,094 | 46.92 |
| OBK | Emu | No | 195,821,276 | 20.87 |
| OBK | GSK | No | 242,085,076 | 25.08 |
| OBK | NIBK | No | 223,266,430 | 23.92 |
| OBK | OBK | Yes | 244,434,188 | 25.24 |
| SBK | Emu | No | 104,537,369 | 13.33 |
| SBK | GSK | No | 125,509,220 | 15.70 |
| SBK | NIBK | No | 115,777,822 | 14.79 |

**Supplementary table S5:** Genome-wide distance matrix calculated using data mapped to the great spotted kiwi. GSK - Great spotted kiwi, NIBK - North Island brown kiwi, LSK - Little spotted kiwi, OBK - Okarito brown kiwi, SBK - Southern brown kiwi.

|  | GBK | NIBK | LSK | OBK | SBK |
| --- | --- | --- | --- | --- | --- |
| GSK | 0 | 0.009082 | 0.00313 | 0.009017 | 0.008918 |
| NIBK | 0.009082 | 0 | 0.00923 | 0.003799 | 0.00513 |
| LSK | 0.00313 | 0.00923 | 0 | 0.009161 | 0.00906 |
| OBK | 0.009017 | 0.003799 | 0.009161 | 0 | 0.004987 |
| SBK | 0.008918 | 0.00513 | 0.00906 | 0.004987 | 0 |

**Supplementary table S6:** Parameters for the hPSMC simulations. GSK - Great spotted kiwi, NIBK - North Island brown kiwi, LSK - Little spotted kiwi, OBK - Okarito brown kiwi, SBK - Southern brown kiwi.

| Species pair | Pre-divergence Ne | Lower limit | Upper limit | Step size |
| --- | --- | --- | --- | --- |
| GSK-NIBK | 36,000 | 1.4M years | 3M years | 200k years |
| GSK-LSK | 16,000 | 200k years | 1M years | 100k years |
| GSK-OBK | 36,000 | 1.4M years | 3M years | 200k years |
| NIBK-LSK | 36,000 | 1.4M years | 3M years | 200k years |
| NIBK-OBK | 17,000 | 200k years | 1.2M years | 100k years |
| LSK-OBK | 36,000 | 1.4M years | 3M years | 200k years |
| SBK-GSK | 36,000 | 1.4M years | 3M years | 200k years |
| SIBK-NIBK | 20,000 | 400k years | 2M years | 200k years |
| SBK-LSK | 36,000 | 1.4M years | 3M years | 200k years |
| SBK-OBK | 20,000 | 400k years | 2M years | 200k years |

**Supplementary table S7:** Percentage of windows and sites resulting in a given phylogenetic topology. Results shown when mapping all kiwi species and the emu to emu assembly assemblies or all kiwi species to the great spotted kiwi reference. gCF - gene (window) concordance factors, sCF - site concordance factors. GSK - Great spotted kiwi, NIBK - North Island brown kiwi, LSK - Little spotted kiwi, OBK - Okarito brown kiwi, SBK - Southern brown kiwi.

| Mapping reference | Emu | | Great spotted kiwi | |
| --- | --- | --- | --- | --- |
| Topology | gCF | sCF | gCF | sCF |
| (OBK, NIBK) | 60.3 | 50.3 | 65 | 57.9 |
| (GSK, LSK) | 91.7 | 83 | 98.9 | 94.9 |
| ((OBK, NIBK), SBK) | 75.9 | 66.9 | 98.9 | 94.9 |
| ((OBK, SBK), NIBK) | 18.5 | 30.3 | 19.4 | 22.9 |
| ((NIBK, SBK), OBK) | 13 | 19.4 | 15.5 | 19.2 |

**Supplementary table S8:** QuIBL results. Total proportion - proportion of windows showing the alternative topology indicated by the columns gene flow pair and outgroup. A difference >10 in BIC scores indicate a significant signal for gene flow. GSK - Great spotted kiwi, NIBK - North Island brown kiwi, SBK - Southern brown kiwi, LSK - Little spotted kiwi, OBK - Okarito brown kiwi. Highlighted rows have a BIC difference > 10 indicating both ILS and gene flow gave rise to the discordant topologies. Total proportion was calculated by dividing the topology count from the total number of windows (763).

| **Species in comparison** | **Gene flow pair** | **outgroup** | **BIC2Dist** | **BIC1Dist** | **BIC difference** | **Topology count** | **Total proportion** |
| --- | --- | --- | --- | --- | --- | --- | --- |
| GSK, NIBK, OBK | GSK-OBK | NIBK | -662.55 | -675.12 | 12.57 | 84 | 0.11 |
| GSK, NIBK, OBK | GSK-NIBK | OBK | -293.98 | -297.80 | 3.82 | 31 | 0.04 |
| GSK, NIBK, SBK | GSK-SBK | NIBK | -910.59 | -919.22 | 8.63 | 109 | 0.14 |
| GSK, NIBK, SBK | GSK-NIBK | SBK | -577.79 | -585.33 | 7.54 | 61 | 0.08 |
| GSK, NIBK, LSK | NIBK-LSK | GSK | -262.93 | -268.04 | 5.12 | 28 | 0.04 |
| GSK, NIBK, LSK | GSK-NIBK | LSK | -243.08 | -247.23 | 4.15 | 27 | 0.04 |
| GSK, OBK, SBK | GSK-SBK | OBK | -694.76 | -707.99 | 13.24 | 69 | 0.09 |
| GSK, OBK, SBK | GSK-SBK | SBK | -625.21 | -630.39 | 5.19 | 66 | 0.09 |
| GSK, OBK, LSK | OBK-LSK | GSK | -279.42 | -285.31 | 5.88 | 30 | 0.04 |
| GSK, OBK, LSK | GSK-OBK | LSK | -286.94 | -292.65 | 5.71 | 31 | 0.04 |
| GSK, SBK, LSK | SBK-LSK | GSK | -271.37 | -275.95 | 4.57 | 28 | 0.04 |
| GSK, SBK, LSK | GSK-SBK | LSK | -291.03 | -298.35 | 7.32 | 31 | 0.04 |
| NIBK, OBK, SBK | OBK-SBK | NIBK | -1634.46 | -1632.10 | -2.36 | 180 | 0.24 |
| NIBK, OBK, SBK | NIBK-SBK | OBK | -1229.77 | -1242.78 | 13.01 | 112 | 0.15 |
| NIBK, OBK, LSK | OBK-LSK | NIBK | -678.15 | -693.29 | 15.14 | 86 | 0.11 |
| NIBK, OBK, LSK | NIBK-LSK | OBK | -284.86 | -287.64 | 2.78 | 30 | 0.04 |
| NIBK, SBK, LSK | SBK-LSK | NIBK | -934.10 | -942.15 | 8.05 | 111 | 0.15 |
| NIBK, SBK, LSK | NIBK-LSK | SBK | -570.60 | -575.53 | 4.93 | 60 | 0.08 |
| OBK, SBK, LSK | SBK-LSK | OBK | -697.70 | -710.33 | 12.63 | 69 | 0.09 |
| OBK, SBK, LSK | OBK-LSK | SBK | -600.91 | -613.32 | 12.41 | 65 | 0.09 |

**Supplementary table S9:** Significances of the *f*-branch results in the form of Z-scores. Z>|3| indicates a significant result. GSK - Great spotted kiwi, NIBK - North Island brown kiwi, SBK - Southern brown kiwi, LSK - Little spotted kiwi, OBK - Okarito brown kiwi. Nan indicates the *f*-branch for said comparison could not be computed due to the topological input requirements of the test and its inability to infer gene flow between sister lineages.

| **Branch descendants** | **LSK** | **GSK** | **SBK** | **NIBK** | **OBK** |
| --- | --- | --- | --- | --- | --- |
| LSK,GSK | nan | nan | nan | nan | nan |
| SBK,NIBK,OBK | nan | nan | nan | nan | nan |
| LSK | nan | nan | 14.72 | 15.85 | 17.04 |
| GSK | nan | nan | 0 | 0 | 0 |
| SBK | 25.91 | 25.41 | nan | nan | nan |
| NIBK,OBK | 0 | 0 | nan | nan | nan |
| NIBK | 0 | 0 | 0 | nan | nan |
| OBK | 9.95 | 9.76 | 10.02 | nan | nan |

**Supplementary figures**


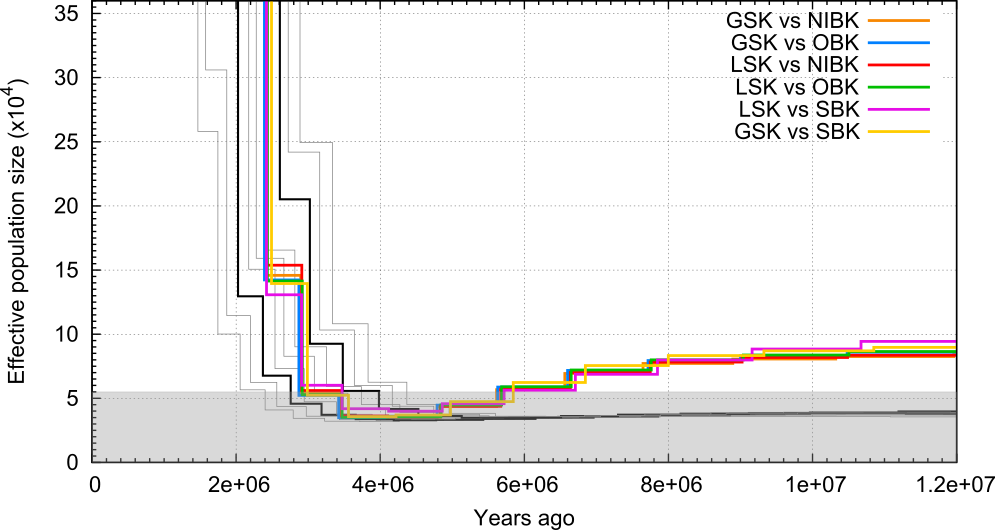


**Supplementary figure S1:** Four pairwise hPSMC comparisons of non-sister kiwi species. Coloured lines show the empirical data. Grey/black lines show the simulated data with the bold line showing the simulations closest to the empirical data. Shaded out area indicates the area <1.5-fold the pre-divergence Ne not considered when determining the simulations with closest fit to the empirical data. GSK - Great spotted kiwi, NIBK - North Island brown kiwi, SBK - Southern brown kiwi, LSK - Little spotted kiwi, OBK - Okarito brown kiwi.


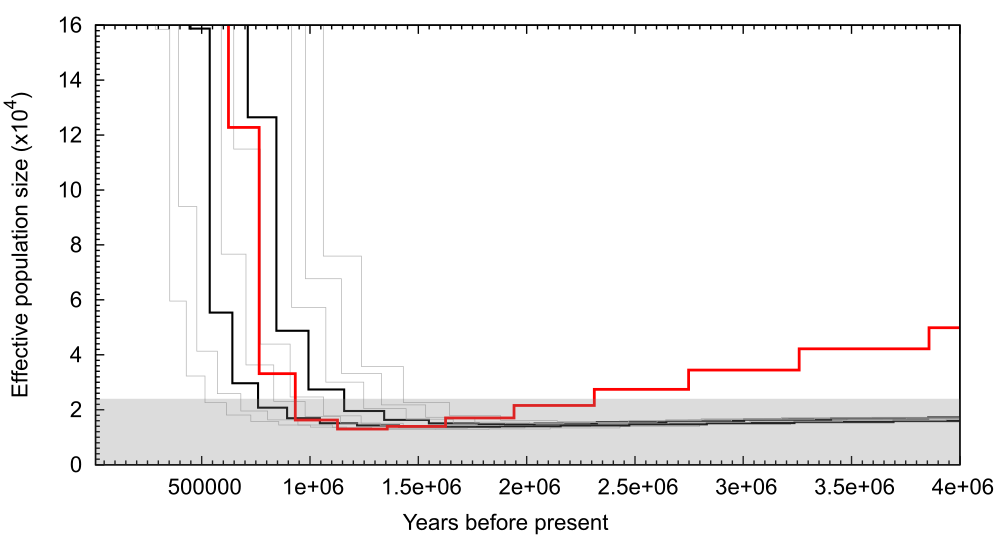


**Supplementary figure S2:** hPSMC comparing the great and little spotted kiwi. Red line shows the empirical data. Grey/black lines show the simulated data with the bold line showing the simulations closest to the empirical data. Shaded out area indicates the area <1.5-fold the pre-divergence Ne not considered when determining the simulations with closest fit to the empirical data.


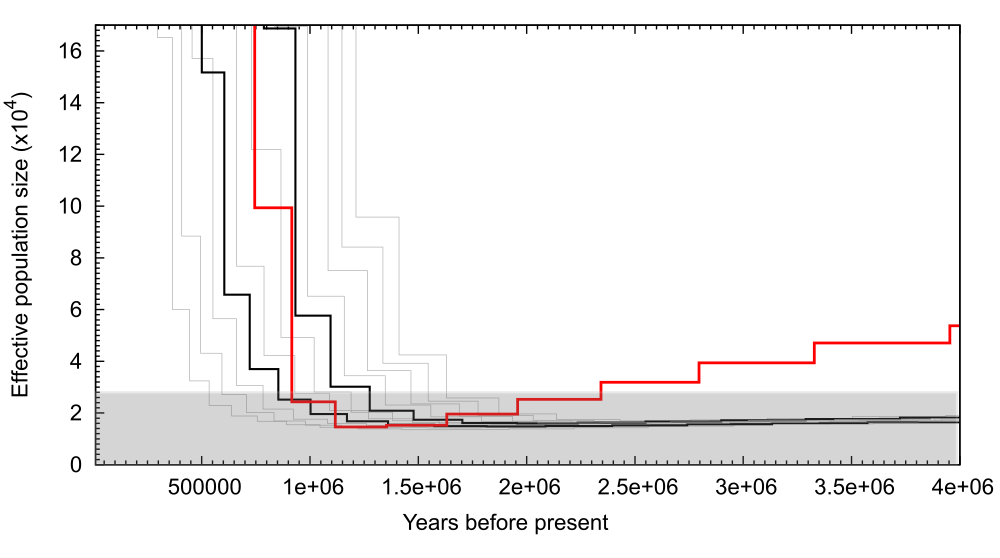


**Supplementary figure S3:** hPSMC comparing the Okarito and North Island brown kiwi. Red line shows the empirical data. Grey/black lines show the simulated data with the bold line showing the simulations closest to the empirical data. Shaded out area indicates the area <1.5-fold the pre-divergence Ne not considered when determining the simulations with closest fit to the empirical data.


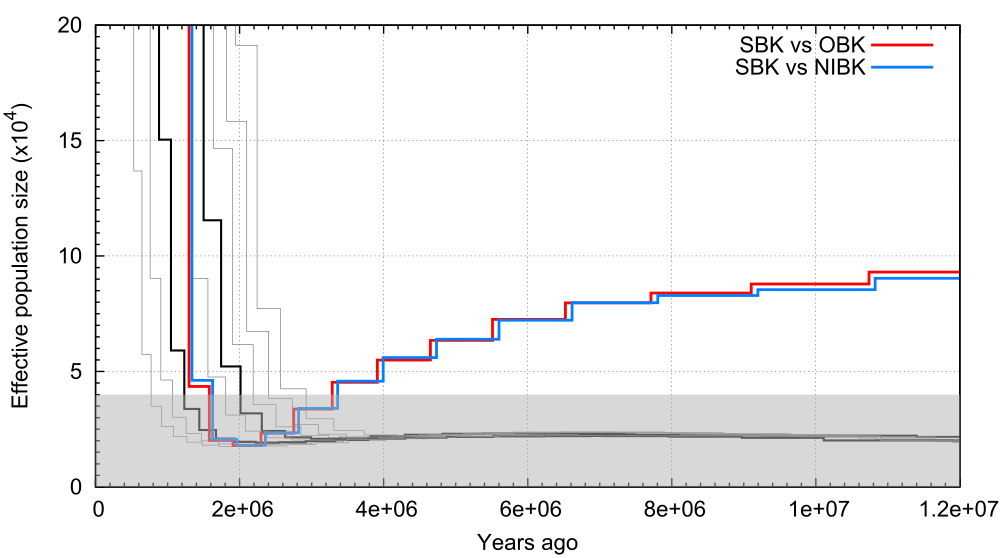


**Supplementary figure S4:** hPSMC comparing the Southern brown kiwi (SBK) to both the Okarito (OBK) and North Island brown (NIBK) kiwi. Red and blue lines show the empirical data. Grey/black lines show the simulated data with the bold line showing the simulations closest to the empirical data. Shaded out area indicates the area <1.5-fold the pre-divergence Ne not considered when determining the simulations with closest fit to the empirical data.

**
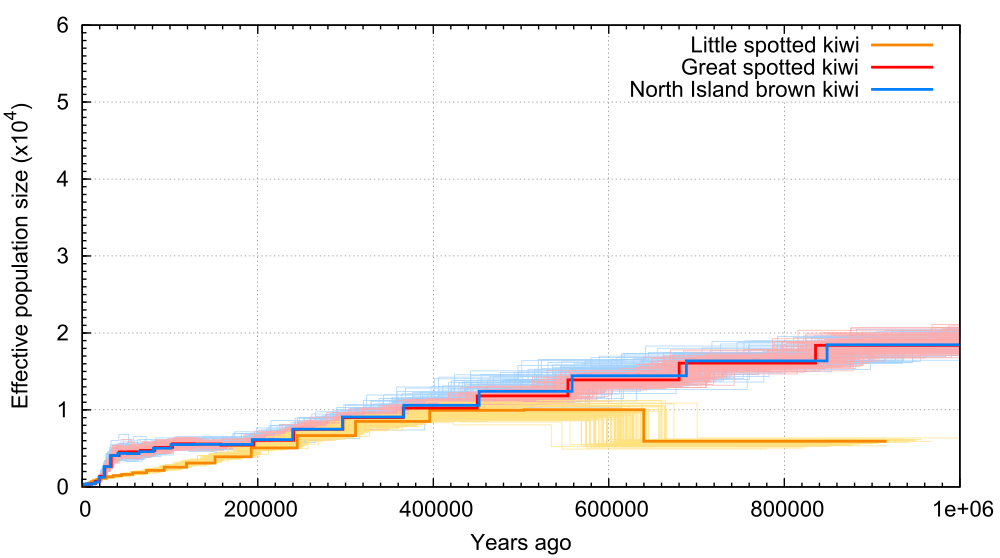
**

**Supplementary figure S5:** PSMC results for the little spotted kiwi based on different mapping references. Colour indicates the mapping reference. Faded lines show the 100 bootstrap replicates produced for each analysis.
