# Supplementary material for: Genomic insights into the evolutionary relationships and demographic history of kiwi
Source: PLoS One. 2022 Oct 10;17(10):e0266430. doi: 10.1371/journal.pone.0266430 (PMC9550048; doi:10.1371/journal.pone.0266430)
Supplement: Attachment — Submitted filename: Response to reviewers.docx [file pone.0266430.s002.docx]

**Response to the reviewers**

Additional Editor Comments:

I have now received reviews from two experts in the field and their reading matches my own. Namely, given that this manuscript is based on publicly available genomic data, the authors will need to put their work in the appropriate context to demonstrate their novel contributions beyond the previously published work. Each reviewer makes additional points of importance with regards to the methodology as well as the interpretation of the results that would also need to be fully addressed. Of particular importance, the authors will need to provide evidence that the inferred model actually matches their data (and that PSMC is able to recapitulate the observations made from the data simulated under the inferred model). In this regard, the authors need to be mindful about the fact that their dataset contains regions affected by direct and linked selection, which can result in serious mis-inference of the population's demographic history (see Johri et al. MBE 2021). To allow the authors to address these comments / concerns, I recommend a major revision.

**Answer:** We thank the editor for their comments and we have addressed all reviewer comments below and think our manuscript is much stronger thanks to the comments. We have now added new details that we think demonstrate some of the novel aspects of the study including more emphasis on gene flow between lineages, older demographic events, and evaluation of results to reference biases. However, we would also like to point out that we chose to submit to PLOS ONE due to the following statement in the journal information: “We evaluate submitted manuscripts on the basis of methodological rigor and high ethical standards, regardless of perceived novelty.” This is because we had already completed the study prior to the publication of Bemmels et al 2021 which generated new genomic data from all kiwi species, heavily taking away from our study. Despite this, we still believe our study contributes to the understanding of kiwi evolutionary history and will be of use for future studies.

Review Comments to the Author

**Reviewer #1:** This project uses previously published genomic data from five species of kiwi birds (and their close relatives, as outgroups) to understand the evolutionary history of this species. The project uses a set of population genomic methods to infer divergence times, introgression history, and inbreeding.

This manuscript has many strengths.

1. I like that the manuscript has a tight focus. While I always enjoy reading a paper that has broad implications, it is also always really nice to read a paper that focuses on the system and the data. It is nicely concise.

2. The study very carefully handles reference bias, which few comparative genomic projects even consider.

3. The figures are generally clear and easy to follow.

**Answer:** We would like to thank the reviewer for their kind words and constructive feedback.

I have a few major suggestions for improvement.

1. There have been two really comprehensive papers published on kiwi evolutionary dynamics already, both out of Jason Weir's research group. This study cites both papers and references them a few times, but I found more should be done here to (1) explain in the introduction how this study can add to these existing studies and (2) compare results found across studies in the Discussion. Especially because the discussion is so focused on the kiwi clade itself, it should more strongly integrate what we already knew about kiwi diversification.

**Answer:** We have now added more details about the previous studies into the introduction and discussion sections.

2. The study uses hPSMC (as I understand it) to infer gene flow between the species. I have never used hPSMC, but I have used PSMC, so I was curious about this application. I read the manual, and I cannot find any evidence that hPSMC allows inference of gene flow. I would recommend the authors either (1) clarify how they modified hPSMC to allow inference of gene flow or (2) clarify the language so that it no longer seems like hPSMC was used for gene flow inference. The discrepancies between divergence times inferred from a phylogeny could be due to ILS, post-divergence gene flow and / or methodological uncertainty. Given the QuIBL results, ILS and / or methodological uncertainty seems most likely.

**Answer:** hPSMC does not specifically infer gene flow events, rather it is a method to infer when populations became completely isolated from one another. This can then be contrasted with other forms of evidence like initial population divergence times to infer how long migration may have occurred after initial divergence. We have now added more details into the methods section about how the method works. QuIBL only looks at the windows that are discordant to the tree topology, but hPSMC uses the whole dataset. We see from site discordance that a lot of the windows that were concordant likely also contained a lot of sites that were discordant. hPSMC is likely picking up the signal from these, giving us the signal of more recent coalescence, likely brought about by gene flow. We have added some more detail into the discussion in regards to this.

3. The study often refers to the importance of ensuring that the reference genome used doesn't introduce bias. Given that this is a recurrent theme in the manuscript, I would suggest discussing this aspect of the study in the introduction and / or discussion to summarize what is known about this issue and why it matters.

**Answer:** We have now mentioned that we test for reference biases at the end of the introduction and added some additional details in the discussion about the impact reference bias can have on gene flow, heterozygosity, and ROH analyses.

MINOR COMMENTS

- L19 - 20: I might be over-interpreting this sentence, but fossils are unlikely to help infer evolutionary relationships -- just evolutionary timings. (See again L61 - 63.)

**Answer:** Here we are referring to the relationships that can be inferred from transitional fossils.

- L58 - 60: "In contrast" -- what are the authors contrasting here?

**Answer:** This has been corrected.

- L82: the Bemmels et al 2021 study also showed this, using whole genome data.

**Answer:** Bemmels et al did show relatively recent effective population declines between 1-10kya in most lineages which is difficult to link to human activity beginning ~800 years ago. Therefore, we specifically chose to report on the use of time sampled individuals as this shows a direct loss of diversity more closely linked to human arrival as opposed to indirectly as with whole genomes from only modern individuals. We have made it clearer that we are specifically referring to human induced range contractions

- L101 - 104: Recommend listing species names here again.

**Answer:** As we only refer to the common names throughout the manuscript, we chose to only mention species names on first mention

- L135: Consider reporting the total number of gene trees inferred.

**Answer:** We have now added in the number of windows remaining after filtering when mapping all data to the Emu and great spotted kiwi.

- L154 - 156: What is the justification for these BIC thresholds?

**Answer:** These BIC thresholds are those specified in the original publication - this has now been referenced.

- L159 - 169: Consider expanding the discussion of the hPSMC approach here. Most readers will be familiar with the PSMC approach, but might not fully appreciate the difference between PSMC and hPSMC.

**Answer:** We have now added more details into the theory behind the hPSMC to this section.

- L171: What is inferred here is technically the substitution rate, no?

**Answer:** It can be difficult to differentiate between the substitution and mutation rates. However, as each individual still contains their own unique mutations that may not become fixed in the population through time and therefore are not species defining substitutions, we chose to use the word mutation rate.

- L199: For this analysis, which variant set was used? The one inferred using ANGSD (L130)?

**Answer:** ROHan does not use *a priori* variant set but instead calculates it from the bam files. We have now clarified this.

- L236: Recommend striking "earliest to diverge" here; see this great blog post here for why: <http://for-the-love-of-trees.blogspot.com/2016/09/the-ancestors-are-not-among-us.html>

**Answer:** Altered the wording to sister to the Okarito brown kiwi, North Island brown clade.

- L237 - 238: 11% and 26% actually not that high! I would recommend reviewing some other phylogenomic discordance papers.

**Answer:** We are referring to high discordance relative to that found in the rest of this particular tree which we have now clarified.

- L241: To my interpretation, the QuIBL results very strongly indicated this is likely just ILS.

**Answer:** That is correct and we have now removed most likely to make it clearer.

- L243 - 253: I find this analysis a bit confusing. The study is comparing divergence times inferred from the phylogeny (which, it would be good to remind the readers here of how these were inferred and how deep they were) to timings inferred from hPSMC? We would expect these two estimates to be very different because they are measuring very different aspects of divergence history. This paragraph makes it clear that this discrepancy could be because of ILS or post-divergence gene flow. Yet, the first sentence of this paragraph seems to suggest this analysis was done to tell us about post-divergence gene flow, but this approach isn't the best approach to study post-divergence gene flow. (Also, another possibility for why these estimates are off: the difficulties in time calibration given uncertainties in substitution rate, fossil dates, and generation time. See also L318 - 320 for the same.) The study appears to be more conclusive that it is post-divergence gene flow in L431, which confuses me.

**Answer:** Here we did not calculate divergence times based on the phylogeny, and we present only the results based on the hPSMC. We have now removed the sentence mentioning their deep divergence times to avoid confusion. We have also reworded to say reproductive isolation instead of post-divergence gene flow. We have also added to the discussion about uncertainties with substitution rates, fossil dates, and generation times. We have also added more details why we think the hPSMC results are more reliable to the start of the discussion.

- L273 - 278: I would consider striking data on the other palaeognath species as they aren't really part of the paper otherwise.

**Answer:** Yes the other palaeognath species data are not utilised in the majority of the manuscript. However, we think it is important to understand the placement of the kiwi diversity within the greater picture. Without the other species we would not know whether the genetic diversity we see can be considered low or not.

- L304 - 306: Can the authors clarify how the hPSMC results show that there was continual gene flow? The high levels of discordance at the site level (Table S6) are in-line with what has been seen in other phylogenomic studies and could simply be the result of ILS.

**Answer:** hPSMC does not necessarily show continual gene flow, more the time period in which 2 genomes have completely coalesced. This has now been described in the methods. We have reworded this section to also mention how the site concordance factors and hPSMC combined show a more complex evolutionary pattern, which could be explained by gene flow post-divergence.

- L322 - L323: High dispersal in birds is one hypothesis for why there is more hybridization, but another hypothesis is that birds seem to evolve postzygotic isolation slowly (Price and Bouvier 2002)

**Answer:** That is an interesting point and definitely something to consider. We have now added this reference to the discussion.

- L327 - L329: One aspect that confuses me here - these species seem to have abutting / overlapping ranges in the present day. Also current ranges do not necessarily reflect historical ranges. So, it is quite possible these species were co-occurring, which would have allowed hybridization pretty easily. And, even with no flight, these birds still disperse? Or is dispersal essentially non-existent?

**Answer:** We have now reworded this part to comment on their overlapping ranges and how that may have aided hybridisation.

- L361 - 3: Here is an example where the Bemmels paper should definitely be cited and used as a comparison point, as they conducted a more formal analysis of the same question.

**Answer:** We have now cited the Bemmels paper which also found the highest diversity in North Island brown kiwi populations.

- L422 - 428: Here is another place where the previous demographic work on kiwi should be cited.

**Answer:** We have now added some information into the previous work on kiwi instead of NZ avian fauna as a whole.

- Figure 1: What is the citation for the historical ranges for these species?

**Answer:** Both the modern and historical ranges are based on the same citation. This has now been made clearer

**Reviewer #2:** In this study, Westbury et al. analyze representative whole genomes from each of five kiwi species to investigate the evolutionary history of this group. They primarily assessed phylogenetic relationships and looked for evidence of phylogenetic discordance due to gene flow and/or ILS, and they inferred historical effective population sizes with PSMC. The authors recover a phylogeny consistent with prior studies, and find some evidence of gene flow and/or ILS, although these latter results are not well resolved. Further, the authors find overall levels of genome-wide diversity that appear consistent with the effective population size histories (Ne of ~25k or less over the past million years), and some ROH, potentially indicative of inbreeding, in the little spotted kiwi.

The study exclusively uses data generated from other studies, which also evaluated the evolution and origins of kiwi. However, these prior studies are not sufficiently acknowledged in this manuscript. The data are not tied to their original sources (Sackton et al. 2019, Weir et al. 2016, Bemmels et al. 2021, more?), and the major findings and unresolved issues from the prior studies are not provided. Further, some important details from the methods are missing, and some of the results appear not to support one another (i.e. the ILS and gene flow analyses). Lastly, some of the conclusions based on the Ne trajectories relating to environmental shifts are unsupported. Given the work that has been done before, I am not sure what the contribution of this study is. In terms of its scope and aims, it is not well differentiated from the previous studies, which are probably more suited to investigating the evolutionary history of this system because they included more data. Overall, the manuscript was relatively clear and the analyses are mostly appropriate, but there are a number of crucial areas that require improvement. What follows is a detailed breakdown of my concerns.

89: The summary of prior genomic studies of kiwi in this introduction is insufficient. As written, the introduction fails to acknowledge that there are a few genomic studies of kiwi already in existence, which also tackle the issues of species origin, population structure, diversity, phylogenetic discordance, etc. with far more samples (e.g. Sackton et al. 2019, Weir et al. 2016, Bemmels et al. 2021). The major findings of those studies and the remaining gaps in knowledge should be presented in the introduction. How the present study addresses those gaps should be stated. How the results from this study agree with, add to, or refute prior studies must also be presented in the Discussion.

**Answer:** We have now added more information to the introduction about the three mentioned previous studies, what they uncovered, and how there are still gaps in the knowledge after the previous studies.

101: Citations are needed for all sequence data and reference genomes used in this study. This information should be added to the methods here and also included in Table S1. Also, in Table S1 the accession codes for the little spotted kiwi are duplicated - typo?

**Answer:** We have added all citations to the main text only to avoid the supplementary table becoming too busy. The accession for little spotted kiwi was a typo. Thank you for catching that.

115: What is the depth of sequencing coverage? This information is essential for assessing the robustness of the methods and results.

**Answer:** We have now added a supplementary table with this information

132: How many sequences were used in the phylogenetic inference? Report sample sizes for all analyses (i.e. for the ILS/gene flow tests as well).

**Answer:** We have now reported the number of windows used in the phylogenetic analyses in relation to the mapping reference.

135: For clarity, specify that the "gene trees" are actually from randomly sampled sequences across the genome, not genes.

**Answer:** We have now used the term “locus trees” to avoid this confusion.

154: Explain the rationale of this BIC cutoff. Is it based on the original QuIBL paper, or used here only?

**Answer:** We have now specified that it is based on the original QuIBL paper.

169, 224: Provide complete details of the PSMC pipeline run parameters.

**Answer:** We have now added the details to the hPSMC section and guide the reader to that section in the PSMC section for parameters.

172: Given the apparent signal of some ILS and gene flow, is this a robust way to estimate mutation rate? Presumably these factors could cause the mutation rate to be under-estimated. Provide a rationale or acknowledgement, and, if possible, compare with other mutation rate estimates from kiwi or similar species.

**Answer:** We have now acknowledged the fact that this could be underestimated due to ILS/gene flow. Our result of 2.0e-8 per generation is comparable to the rate from Bemmel et al 2021 which estimated a rate of 1.34e-8 per generation, which we have now mentioned in the methods.

204: Are the kiwi genomes highly contiguous? How does contiguity of the assemblies potentially impact ROH identification? Add more information to the text or supplement about the contiguity of the genome assemblies.

**Answer:** We have now added a supplementary table with the assembly statistics for the assemblies used in this study. All contain N50 >1Mb and there appears to be no obvious effect of N50 on % of ROH

209: Clarify if the values in Fig. 2B were obtained from ROHan with ROH length set to 1 Mb for all species.

**Answer:** We present the results from the three different window sizes in Fig 2B which has now been clarified in the figure legend.

220: What is meant by "this species"? This part is unclear.

**Answer:** This was referring to the little spotted kiwi, which we have now clarified

225: Clarify that the PSMC results were checked for overfitting (did all intervals contain >=10 recombinations, as recommended by <https://github.com/lh3/psmc>?).

**Answer:** Yes we did check for this and it has now been added.

237-241: A graphical representation of the gene flow and demographic history results should be provided, if possible. It's hard to grasp these results from the text only. The authors could look to Edelman et al. 2019 for inspiration. More detailed demographic model figures are standard in the literature, and very helpful for understanding the results, but other figures to visually show the amount of gene flow/ILS could also be used.

**Answer:** It is difficult to represent our results in a single figure as the hPSMC suggests there was gene flow between all lineages after their divergences. As we have now added to the discussion, ILS being the only reason behind discordances (based on the QuIBL results) is rather unlikely. We have therefore decided to not include a graphic in this case.

264-271: Is the aim to look at "recent" inbreeding? How recent? Be explicit. Length of ROH corresponds with the number of generations back to the shared common ancestor. ROH <=1 Mb can originate from shared ancestors many generations ago, which may or may not be considered "recent" depending on the context, and might not even be reflective of "inbreeding" per se, but rather of limited population size. Given that the other demographic analyses deal with far more ancient history tens to hundreds of thousands of years ago, while some of the species declines occurred far more recently in the post-colonial era within the past thousand years, the communication of these results needs to be more precise.

**Answer:** We have now added information to the methods section about what the ROH represents in terms of number of generations since inbreeding. Depending on the length of ROH this changes how long ago inbreeding may have occurred. In the methods section we calculate how the length of ROH corresponds to the number of generations since inbreeding based on two recombination rates. ROH>0.5Mb equates to inbreeding occuring within the last 47.6 or 33.3 generations, ROH>1Mb equates to inbreeding occuring within the last 23.8/16.6 generations, and ROH>2Mb equates to inbreeding occuring within the last 11.9/8.3 generations. If we assume a generation time of 25 years, this means that our 2Mb ROH estimates mean inbreeding occurred within the last ~250 years.

273: It would be helpful to add the other species to this figure.

**Answer:** For the ROH we chose to only focus on the kiwi as that is the focus of the manuscript. Therefore we chose to leave the other non-kiwi species in the supplementary material.

216-22 and 281-286: Why is there a "bias" for these particular genomes when aligning to a conspecific reference? Does it have to do with the same/different individual being used for the resequencing and the assembly? This is unclear. Explain.

**Answer:** This is a good question and one that is very difficult to answer without generating new genome assemblies. In Prasad et al, it was seen that when the raw reads from the Okarito brown kiwi we reassembled with a different assembly software (SOAPdenovo2 vs the published ALLPATHS-LG assemblies) the results become overall more in line with what would be expected based on results mapping to assemblies from two other kiwi species, suggesting something was wrong with the assembly itself. However, the new assembly was much more fragmented, limiting its ability to perform other analyses such as ROH.

303-308: These results are not consistent. The QuIBL analysis suggests there is little evidence of gene tree discordance overall, consistent with some ILS, but the hPSMC results suggest continuous gene flow. Later, "long-term" ILS is mentioned as a possibility (line 319), which would be surprising given the relatively limited population sizes of these species (~20k or less under the assumed mutation rate). A more coherent explanation of the results is needed in order to tie them together and support the study conclusions. I might also suggest using additional methods to address these issues, since the analyses included in this paper do not seem sufficient or convincing.

**Answer:** We have now rewritten this paragraph to discuss why there could be discrepancies between the QuLBL and hPSMC results. In brief, QuIBL looks at ILS/gene flow causing discordant locus trees but not taking into account the sites within said locus/window. hPSMC on the other hand is not limited by this. As we see in the site discordance factors, there are a lot of sites supporting alternative topologies relative to locus trees, which also lends support toward our hPSMC rather than QuIBL results.

360-361, 365: Species with long generation times will not necessarily show signals of recent bottlenecks in genome-wide heterozygosity. This study does not adequately resolve whether the observed ROH are due to very recent inbreeding or more distant common ancestry. These levels of genome-wide diversity in the kiwi genomes appear largely consistent with the long-term Ne values in each species, which is not surprising. These points need to be made clear.

**Answer:** See above comment about ROH and number of generations since occurrence

414-421: It is well known that PSMC provides effectively no resolution for this time period (25 kya to present). Furthermore, inspection of Fig. 3 does not appear to show a marked and consistent signal of decline among all species. This is mostly speculation and should either be rephrased to avoid misleading the reader, or removed.

**Answer:** We have now rephrased the start of this paragraph and added a cautionary statement about PSMC in more recent time periods.

435-437: The results presented in this study are insufficient to support this claim. As stated above, there is not enough power in the analyses of these single genomes to demonstrate the impact of volcanism over other forces. Furthermore, linking PSMC curves to environmental shifts is dubious, since PSMC analyses involve several over-simplifications and assumptions. For example, the results are highly dependent on the assumed mutation rate and generation time, which are often unknown (in this case, we don't know how reliable the mutation rate is).

**Answer:** We have now removed the conclusions section
