# Supplementary material for: Genomic insights into the evolutionary relationships and demographic history of kiwi
Source: PLoS One. 2022 Oct 10;17(10):e0266430. doi: 10.1371/journal.pone.0266430 (PMC9550048; doi:10.1371/journal.pone.0266430)
Supplement: Attachment — Submitted filename: Reviewer responses.docx [file pone.0266430.s003.docx]

Additional Editor Comments:

There remains a major concern about the reliable distinction of incomplete lineage sorting from post-divergence gene flow in this study. Currently, the authors favor one process over the other, without much convincing evidence. Additional analyses will be needed to address this issue (as well as several smaller ones highlighted by the two reviewers). Both reviewers agree that the writing itself needs to be improved as well, in particular with regards to the distinction between previously published results and novel insights from this study (which was already pointed out in the first round of reviews) and the discussion (which needs to be more focused). For reproducibility, a permanent repository for any scripts used in the analyses should be included in a future revision.

**Answer:** We thank the editor and the reviewers for their comments. We have now changed the window size for the phylogenomic gene flow analyses and included a new analysis to test for gene flow in the form of the *f*-branch statistic. With these new additions, it is much clearer that the evolutionary history of kiwi consisted of post divergence gene flow. We have also substantially changed the discussion section and added subsections to ease reading. Finally, we have uploaded example commands and scripts required for all analyses onto Github for reproducibility.

Reviewer #2: I have read the revised manuscript from Westbury et al. and find that although it has improved, there are outstanding issues. I am mostly satisfied that the study has been differentiated from already published works. However, my biggest concern is that the conclusions about incomplete lineage sorting (ILS) versus post-divergence gene flow are not supported by the evidence. Also, a couple of minor issues I mentioned in my original review were not adequately addressed. See below for complete details. I recommend further revision before the manuscript can be accepted for publication.

ILS/gene flow analyses: The presentation of the ILS/gene flow results are slightly more clear now, and after some further thought, I am not convinced that the results favor post-divergence gene flow over ILS. First, hPSMC simply does not distinguish between these two processes, so it is not sufficient evidence either way. Second, QuIBL is supposed to specifically differentiate between ILS+gene flow and ILS alone, and the authors simply chose to disagree with the QuIBL results tending to support the ILS alone model. The argument of site discordance within congruent windows suggesting post-divergence gene flow does not make sense to me. What seems to be happening is that kiwi diverged very recently, and may really be incipient species or even subspecies that are still capable of hybridizing. Kiwi have long generation times, apparently only diverged within the last few million years, and it is well known that bird genomes evolve slowly. Under this scenario, I would expect ILS to be more of a factor than post-divergence gene flow. In my original review, I suggested performing additional analyses to better resolve this question. I reiterate that suggestion here. One possibility might be to use the ABBA/BABA test, but there are probably other options as well.

**Answer:** Due to the high levels of discrepancies between our gene and site concordance factors we reran the analysis with smaller windows (2kb instead of 20kb) to ensure we are only sampling a single loci in each window. The QuIBL results now show a mixture of both ILS and gene flow explaining the discordances. We have now also run the *f*-branch statistic which is similar to an extended version of the ABBA/BABA test, allowing for correlations between shared alleles to be taken into account in interpretations. The *f*-branch also supports our finding of gene flow between kiwi lineages with overall similar results to the QuIBL analysis.

Discussion: At about four pages, the Discussion is far too long. Compare with the results, which are just over one page in length. The Discussion needs to be condensed, and the main conclusions that are supported by the analyses in the paper should be stated clearly. As is, the Discussion is meandering, not very well organized, and hard to digest.

**Answer:** We have now added subheadings into the discussion to guide the reader. We have also streamlined the content but with the addition of the new analyses it is still somewhat long**.**

115-123: Briefly mention the mean or range of sequencing depths.

**Answer:** We have now added a section about the mapping results to the results including a mention of the genome-wide coverages.

165: I don't advocate for using the term "locus trees" instead of "gene trees". Gene tree is the terminology used in the literature. It would be better to simply state, once, that the "gene trees" are actually just from windows, not genes.

**Answer:** We made this change to appease a previous reviewer but have now reverted back to gene tree and comment that this refers to each window at the first mention of the term in the methods

Table S1: The previously published data (reads, assemblies) should have citations for sources. A supplemental table being too "busy" isn't really justification for failing to give proper attribution and accurately document where the data came from.

**Answer:** We had fully documented this in the main manuscript under the methods section but have now added the reference number of the associated study to the supplemental table S2.

Reviewer #3: In this manuscript, the authors re-examine the evolutionary relationships between kiwi species and look for introgression between these species. I like the introduction as well as the figures and see value in publishing confirmatory results besides clear values of nucleotide diversity (for instance in Figure 2). However, as most of their results are confirmatory, which results are confirmatory and what has been gained from performing this study should be made explicitly clear. Here are my more specific comments:

1. Please provide a link (at Github or Dryad etc) to the scripts used to perform the analyses in this study.

**Answer:** We have now generated a github page with examples and the necessary scripts for the analyses used in this manuscript. It can be found at https://github.com/Mvwestbury/Kiwi-genomes

2. In the abstract, the authors claim that they uncover similarities and differences in the demographic histories of the kiwi species studied here. However, as Weir et al (2016) already uncovers similarities in the demographic histories using PSMC, the authors should mention that they confirm previous observations of past population size changes. If the authors disagree with this, then they should mention in the abstract what they uncovered that is different.

**Answer:** Weir et al (2016) utilised SNP data from GBS and PopSizeABC to make inferences about population size through time. PSMC requires full genomes which were not available at the time. However, Bemmels et al 2021 did have genomes available and were able to look into the similar time frames to us. We have therefore slightly reworded the abstract to focus more on the hypotheses on what could have caused the similarities and differences rather than the uncovering of the similarities and differences themselves.

3. Overall, I really like Figure 2. Could you perhaps also provide the synonymous site heterozygosity or heterozygosity calculated only using 4-fold degenerate sites as well? I suggest that as it may be helpful later for other studies.

**Answer:** Unfortunately, there are no gene annotations available for all kiwi genomes (apart from the North Island brown kiwi), preventing us from doing these analyses.

4. Lines 347-360: We now know from multiple studies that unaccounted for factors such as the presence of purifying selection across the genome (Johri et al 2021) and hidden substructure in populations (Chikhi et al 2018) can result in biased inference of population history when using MSMC/ PSMC -like approaches. Although there has been no thorough investigation of how hPSMC would be biased by the same factors, it most likely would. Thus, the authors should discuss how this could affect their results.

The same reasons might be responsible for observing similar temporal patterns of population decline across all five species, as mentioned in lines 491-493.

**Answer:** We have now added some sentences as to why we think our hPSMC results are not biased by population structure and/or negative selection to the results section. We have also mentioned how a decrease in Ne could be due to population size or population connectivity decreases in the discussion.

5. This is simply a suggestion, and the authors can decide whether they like it or not. You could place the section about mapping to different reference genomes and obtaining the same species tree in the Results section (instead of the Methods section), if you like, as I believe that it is an interesting result. However, this is entirely up to the authors.

**Answer:** We have now moved the section about species tree and reference from the discussion into the results

6. The discussion section currently meanders a bit. Subsections with headings will really help the reader get the main points from the discussion.

**Answer:** We have now added subsections to try and help the reader.

Minor Comments:

1. Lines 368-370: could you please refer to the respective figures from your manuscript.

**Answer:** We have now moved this paragraph to the results section

References:

Chikhi L et al. 2018. The IICR (inverse instantaneous coalescence rate) as a summary of genomic diversity: insights into demographic inference and model choice. Heredity. 120:13–24.

Johri P et al. 2021. The impact of purifying and background selection on the inference of population history: problems and prospects. Mol. Biol. Evol. 38:2986–3003.
