# Supplementary material for: Genomic insights into the evolutionary relationships and demographic history of kiwi
Source: PLoS One. 2022 Oct 10;17(10):e0266430. doi: 10.1371/journal.pone.0266430 (PMC9550048; doi:10.1371/journal.pone.0266430)
Supplement: Attachment — Submitted filename: Reviewer responses.docx [file pone.0266430.s004.docx]

Reviewer responses.

Thank you for submitting a revised version of your manuscript to PLOS ONE. Most of the reviewers' comments were addressed in this revision, however there remain a few outstanding points that should be addressed: 1) minor modifications in the text would help to improve clarity and 2) p-values should be included in Table S9 to strengthen the analysis**.**

**Answer:** We have added a point by point response to the outstanding comments of the reviewers. In summary to the specific points mentioned by the editor: **1a)** We have slightly modified the *Interspecific evolutionary relationships* results section to make it clearer about how we are addressing potential reference bias. **1b)** We decided against reviewer 2’s suggestion of cutting the discussion text about the limitations of the Fbranch test and how that can influence the interpretations of results as we think it is important to discuss these. The aim of the more detailed discussion here is to prevent misinterpretations based on taking the results at face value. **2)** We assume that when the editor/reviewer refers to p-values they actually mean Z-score. P-values are not available with the software used by Z-scores also present the statistical significance with |Z|>3 indicating a significant result. There are no Z-scores available because the comparisons can not be made between the lineages in question due to the topological requirements of the test and inability to test for gene flow between sister lineages. In regards to points **1b** and **2**, a lack of familiarity with the Fbranch method employed likely led to these concerns and we hope our explanation has made it clearer as to why we presented our results the way we did.

Reviewer #2: I thank the authors for addressing my previous comments. I have a few more relatively minor concerns with regard to some of the changes in the revised manuscript, as detailed below.

26-27: Would help to state which species this is referring to, or what the exceptions are.

**Answer:** Done

188: Define fb

**Answer:** This was defined in the figure 2 legend but we have now added a definition to the text.

189-190: Blocks of what size were used?

**Answer:** The genome was split into 20 blocks as per the default parameters. We have now mentioned this in the methods

307-311: These sentences are not sufficiently clear: 307: Similarities between what? the ingroup and outgroup? Is the bias in the emu's diversity, or the kiwi's? 310: Why is the bias caused by using a conspecific reference as opposed to a heterospecific reference? You don't actually know which produces the more accurate result.

**Answer:** We are referring to similarities between the ingroup and outgroup as we wrote “inflating signals of genetic similarities between a highly divergent outgroup and an ingroup species used as mapping reference”. However, we have now included “by the emu artificially appearing more genetically similar to the great spotted kiwi than the non-reference species.”

We think there has been some misunderstanding by the reviewer here. This is not in relation to the diversity estimates but to the phylogenetic inferences as it is under the “Interspecific evolutionary relationships” subheading.

The problem is not caused by mapping to a conspecific reference but a heterospecific one which we are doing in this analysis for 5 out of the 6 individuals. For example, If we were to map the outgroup (emu) to an ingroup (kiwi) the reads more similar to that kiwi species are more likely to successfully map. Due to the higher levels of perceived similarities between the reference and the outgroup, the reference may in turn look less like the other kiwi species and more like the emu. This has been tested and shown in the paper that we refer to [41] Liu S, Westbury MV, Dussex N, Mitchell KJ, Sinding M-HS, Heintzman PD, et al. Ancient and modern genomes unravel the evolutionary history of the rhinoceros family. Cell. 2021;184: 4874–4885.e16.

The reviewer is correct that we do not know which is the more accurate result. However, since regardless of reference we see more or less the same result, we know that reference genome selection is not a problem in our results and can therefore be more confident in them. We mention this in the section “Regardless of the genome assembly selected as mapping reference, the vast majority of windows in our phylogenomic analysis support”

We have slightly modified this section to make it clearer.

310: Fewer not less

**Answer:** Corrected

412-423: I suggest removing this part of the Discussion. It's very hard to comprehend and is better understood by visual by examination of the figures. Obviously the specifics are complicated and there are signals of gene flow/ILS throughout the phylogeny, so I recommend just summarizing with the big picture: there are signals of gene flow and/or ILS throughout the tree, which is not too surprising for a very young radiation like this. I still think the analyses of gene flow v. ILS are a little shaky, and deeper investigations of particular species histories should be left to future studies with larger datasets (ie, more individuals).

**Answer:** We would prefer to keep this in the discussion as it helps to explain some of the patterns we see in our results that do not make biological sense. We added the extra panel in figure 2 (C) to be able to guide the reader in the interpretations as we assume the reader will look at the figure when it is referred to in the text. We do not want the reader to take the results at face value as this could lead to misinterpretation. We instead prefer to present the limitations of the analysis and discuss them in this section.

Fig 2: All cells of the matrix are a uniform shade of grey, so the results cannot be seen. Also, what is the basis for the gene flow events "hypothesized" by the authors? Explain this, or, preferably, eliminate and just show the actual results.

**Answer:** How we devised the hypothesised events are heavily detailed in the discussion section. We think that removing them here would remove an important piece of our interpretation and make it more difficult for the reader to follow which events we are referring to in the discussion. The grey indicates comparisons that could not be made which we now mention in the figure legend.

Table S3: Define N50, L75

**Answer:** Added

Table S8: "production" should be "proportion" ?

**Answer:** Corrected

Table S9: I have not used this method of calculating f-branch statistics, but the fact that no p-values could be computed for about half the table is very disconcerting. What is the explanation? Have the authors made an effort to rectify this? I consider this a serious flaw with this analysis.

**Answer:** We had mentioned in the table legend that “Nan indicates the *f*-branch for said comparison could not be computed”. However, we have now added “due to the topological input requirements of the test and its inability to infer gene flow between sister lineages” to make it clearer that it is not a lack of Z-value but a lack of being able to do the comparison at all.

Reviewer #3: The authors have addressed most of the comments.

The github link is not really working and so the data availability could not be verified.

**Answer:** The github link github.com/Mvwestbury/Kiwi-genomes should be working. We have also now added the fixed DOI <https://doi.org/10.5281/zenodo.7099703> that we have checked is also working
